# An Appreciation of "The Hospital."

**Published:** 1896-03-28

**Authors:** T. E. Hayward

**Affiliations:** Offices of the Medical Officer of Health, Haydock, St. Helens.


### Editor's Letter-Box.

[Our correspondents are reminded that proxility is a great bar to publication, and that brevity of style and conciseness of statement greatly facilitate early insertion.]

### AN APPRECIATION OF "THE HOSPITAL."

Sir,—May I be permitted to forward to you herewith enclosed a copy of my annual report for the year 1895? For the favourable notice which you have thought fit to accord to my two preceding annual reports I have to thank you, not so much personally as on behalf of the large number of those who, like myself, have to combine the duties of medical officer of health with the arduous and distracting work of general practice. The general practitioner, having to do something of everything, in presence of the high standards of attainment which now exist in every special department of medical work, cannot but feel "who is sufficient for these things?" This report, such as it is, is the attempt of a general practitioner to follow as best he may along the lines laid down by "specialists" in the department of public health. In common, doubtless, with many others I have felt helped and encouraged in my work by the way in which you have always upheld the general practitioner, and amid local discouragements I have often felt inspired by the articles in which you showed how fully you understood and sympathised with the difficulties of the often-despised general practitioner. I see that of late some doubt has been insinuated as to whether The Hospital is really a "medical paper." As far as I am concerned I find in it more help than from those journals which take to themselves such exclusive rights. There is a refreshing reminder of bracing breezes and bright sunlight, instead of the somewhat dim and musty associations of the older established papers.—I am, Sir, yours faithfully,

T. E. Hayward.

Offices of the Medical Officer of Health, Haydock, St. Helens.

